# Small intestinal strictures as a complication of mesenteric vessel thrombosis: two case reports

**DOI:** 10.4076/1752-1947-3-8623

**Published:** 2009-09-01

**Authors:** Sandeep Patel, Shashank V Gurjar

**Affiliations:** 1 Medway Maritime Hospital, Gillingham, Kent, UK

## Abstract

**Introduction:**

Small intestinal strictures secondary to mesenteric vessel thrombosis are a rare entity and thus often result in delayed diagnosis. We present two cases of ischaemic small bowel strictures secondary to mesenteric vessel thrombosis, and describe how they were subsequently managed.

**Case presentation:**

We present two cases of abdominal pain, one acute and one chronic, in which the eventual diagnosis was of bowel strictures secondary to arterial and venous vessel thrombosis. In both patients, a Caucasian male aged 67 and a Caucasian female aged 78, the diagnosis was delayed because of the infrequency of their presentation. Both patients eventually underwent a resection of the affected portion of bowel with primary anastamosis and made uneventful recoveries.

**Conclusion:**

There are multiple medical and surgical management options for small bowel strictures and these depend on the aetiology of the stricture. Ischaemic small bowel strictures represent a difficult diagnosis and the potential resulting delay may be partially responsible for increased morbidity. Barium small bowel follow-through should be used in making the diagnosis.

## Introduction

Small intestinal strictures secondary to mesenteric vessel thrombosis are a rare entity and thus often result in delayed diagnosis. The patient often presents with chronic bouts of abdominal pain associated with symptoms of intermittent small bowel obstruction. Results of routine investigations are often normal and subsequently diagnosis and often treatment can be difficult. We present two cases and highlight possible investigative and management strategies.

## Case presentation

### Patient 1

A 67-year-old Caucasian man presented acutely with a 5-day history of recurrent bouts of epigastric pain, nausea and vomiting. His previous surgical history included coronary artery bypass surgery, appendicectomy and open cholecystectomy with subsequent surgery for recurrent incisional hernia. No personal or family history of thrombosis-related conditions was given. On examination, he had low grade pyrexia but was cardiovascularly stable. Abdominal examination revealed mild distension with tenderness in the epigastrium and right hypochondrium.

**Figure 1:**
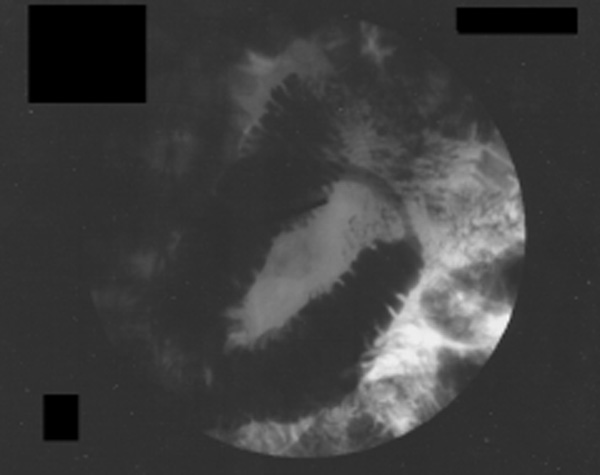
**Small bowel follow-through showing a smooth stricture with proximal small bowel dilatation**.

Initial investigations revealed leucocytosis and a raised C-reactive protein level. He underwent medical review to rule out a cardiac aetiology (normal electrocardiogram (ECG), negative troponin test). An abdominal ultrasound showed no abnormality. He was discharged with out-patient follow-up but continued to experience intermittent abdominal pains. A subsequent computed tomography (CT) scan revealed a small hiatus hernia, and thickening of the distal (third part) duodenum. On endoscopic visualisation, no abnormality was demonstrated. Eventually, a small bowel follow-through was performed (Figure 1): this demonstrated a short smooth stricture in the upper part of the small bowel with mild proximal dilatation. Radiological findings were suggestive of a possible Crohn's stricture or an underlying malignancy. At laparotomy, a stenosing segment of the mid-jejunum and a chronically thickened, dilated proximal gut were found. The stenosing segment was resected and a primary side-to-side anastomosis was fashioned using a GIA-75^®^ stapling device.

Macroscopic examination of the resected specimen showed an area of ulceration and haemorrhage measuring 50 mm in length. Histology tests confirmed a deep chronic ulcer, granulation tissue and marked fibrosis disrupting the muscularis propria; mesenteric sections revealed organised thrombosis of medium and small vessels with mild to moderate recanalisation. A thrombophilia screen was found to be unremarkable. On the advice of the haematologist, the patient was commenced on warfarin and made an uneventful postoperative recovery; to date, he has experienced no relapse of his symptoms.

### Patient 2

A 78-year-old Caucasian woman presented with sudden onset abdominal pain with associated nausea and vomiting. She had a past medical history of a myocardial infarction, chronic obstructive pulmonary disease (COPD), transitional cell carcinoma of the bladder and an appendicectomy as a child.

**Figure 2:**
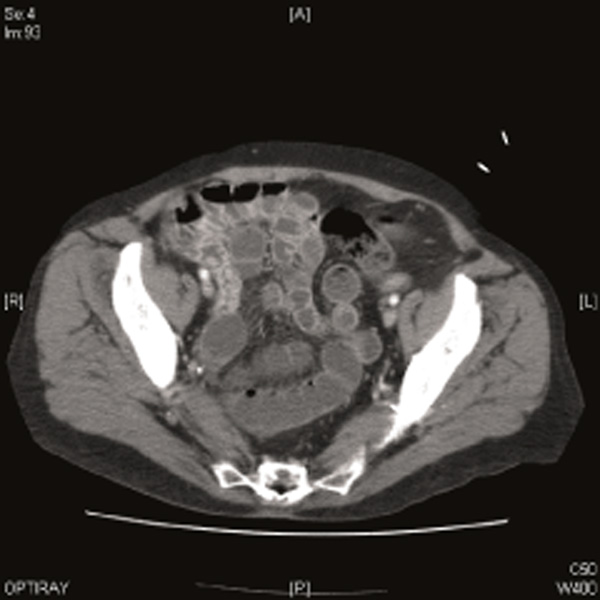
**Computed axial tomography scan showing thickening of the terminal ileum with intramural gas**. Of note, no definite stricturing can be seen on this slice.

On examination, she was tachycardic with evidence of marked lower abdominal peritonism. Blood tests showed neutrophilia and mild acidosis; plain film radiology was diagnostically unhelpful. An ECG was obtained which showed ventricular ectopics and changes suggestive of lateral ischaemia: on cardiological review, these changes were consistent with an old infarct and troponin T levels at 12 hours were negative. A CT scan revealed a probable left ventricular aneurysm and evidence of a thickened loop of terminal ileum containing intra-mural gas, suggestive of ischaemia (Figure 2). A pre-operative echocardiogram confirmed a moderately sized apical ventricular aneurysm containing a small mobile thrombus.

At laparotomy, a 25 cm segment of strictured ischaemic terminal ileum was found with a 5 cm area of necrotic bowel contained within it. This segment was resected and primary side-to-side anastomosis was undertaken using a TLC-75 stapler. The patient returned to the high dependency unit (HDU) and made an uneventful recovery. Results of a thrombophilia screen were negative and histological examination revealed intermittent full thickness necrosis with an atrophic and chronically inflamed mucosa consistent with the effects of chronic ischaemia. No venous thrombosis was seen. The patient made an uneventful recovery and was discharged 5 days later.

## Discussion

Small bowel strictures associated with mesenteric vessel thrombosis are an uncommon entity, having been reported infrequently in the literature [1-4]. In an experimental study which looked at the effects of deliberate embolisation of a primary branch of the superior mesenteric artery in dogs, a local stricture was noted to develop within the embolised segment of the intestine [5]. Mesenteric thrombosis is generally associated with haematological abnormalities that encourage the pro-thrombotic state (for example, protein C or S deficiency) [6]. The development of ischaemic strictures has also been associated with pancreatitis [7], Buerger's disease [8], thromboangiitis obliterans [9] and blunt abdominal trauma [10]. An ischaemic stricture of the proximal jejunum was noted in a nine-month-old baby with atypical Kawasaki disease who presented with fever and coronary artery aneurysms [11]. Hypotensive drugs have also been implicated in intestinal ulceration and stricture formation [12].

In cases of chronic bouts of abdominal pain associated with symptoms of intermittent small bowel obstruction, diagnosis can be difficult. The differentials include Crohn's disease, lymphoma, carcinoid infiltration, ischaemia, tuberculosis and radiation enteritis. Blood tests, routine radiological imaging or endoscopy may prove negative. A barium small bowel follow-through study can be the most useful test. A smooth uniformly narrowed segment of small bowel may be seen with evidence of proximal dilatation. An ischaemic stricture develops as an end result of inflammation and scarring, leading to rigid thickening and fibrosis of the locally involved bowel wall musculature. When the progress of a normal peristaltic wave is impeded, obstruction may ensue.

The clinical management of such strictures is dependent on the diagnosis. Formal histological confirmation has to be sought so that the appropriate treatment can be commenced. Intervention is only necessary when an individual is symptomatic. Steroids, aminosalicylic acid preparations and immunomodulators have been used as part of the medical management of active Crohn's disease; surgical treatment options include endoscopic balloon dilatation to relieve the obstruction; strictureplasty and formal resection of the affected segment.

The most important diagnosis to rule out in patients with small bowel strictures is malignancy. Although in these two patients, the diagnosis is of a stricture of vascular origin, small bowel strictures should be considered malignant until shown to be otherwise. In our patients, preliminary investigation by way of computed tomography scan and gastrointestinal endoscopy had shown this not to be the case and they were managed appropriately.

The current literature on the management of malignant small bowel strictures is limited and thus management is complicated. Cases where malignant strictures are strongly suspected or proven should be managed by specialists in specialist centres.

## Conclusion

A greater awareness of the association between mesenteric thrombosis and intestinal strictures would reduce the delay in diagnosis and subsequent treatment. Careful CT scan interpretation may be of benefit but in this light, the barium small bowel follow-through is the investigation of choice.

## Abbreviations

COPD: chronic obstructive pulmonary disease; CT: computed tomography; ECG: electrocardiogram; HDU: high dependency unit.

## Consent

Written informed consent was obtained from the patient for publication of this case report and any accompanying images. A copy of the written consent is available for review by the Editor-in-Chief of this journal.

## Competing interests

The authors declare that they have no competing interests.

## Authors' contributions

SP collected data, wrote and edited the manuscript. SG was involved in the patients' care and wrote and edited the manuscript. Both authors read and approved the final manuscript.
